# Supplementary material for: Scaling of organ masses in mammals and birds: phylogenetic signal and implications for metabolic rate scaling
Source: Zookeys. 2020 Nov 2;982:149–59. doi: 10.3897/zookeys.982.55639 (PMC7652810; doi:10.3897/zookeys.982.55639)
Supplement: Supplementary material 1 — Figures S1–S5. Additional graphs with result analysis and phylogenetical trees used in data analysis [file zookeys-982-149-s001.docx]

**Supplemental material**

**Phylogenetic trees of studied birds and mammals**


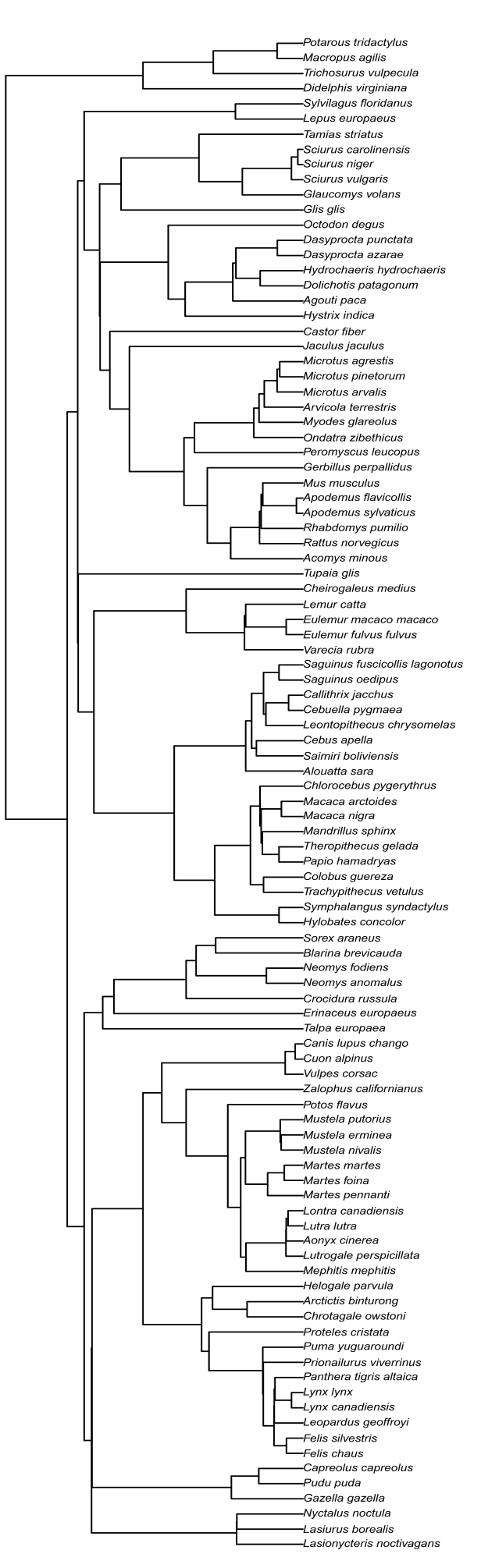
Phylogenetic tree for mammals

Figure S1. Phylogenetic tree of mammal species used in phylogenetically informed analysis. After: [Navarrete, van Schaik and Isler (2011](#_ENREF_22)).

**Phylogenetic tree for birds**

Fig. S2. Phylogenetic tree of bird species used in phylogenetically informed analysis. Built with the data from Tree of Life: http://tolweb.org/tree/.

**Organ mass scaling**


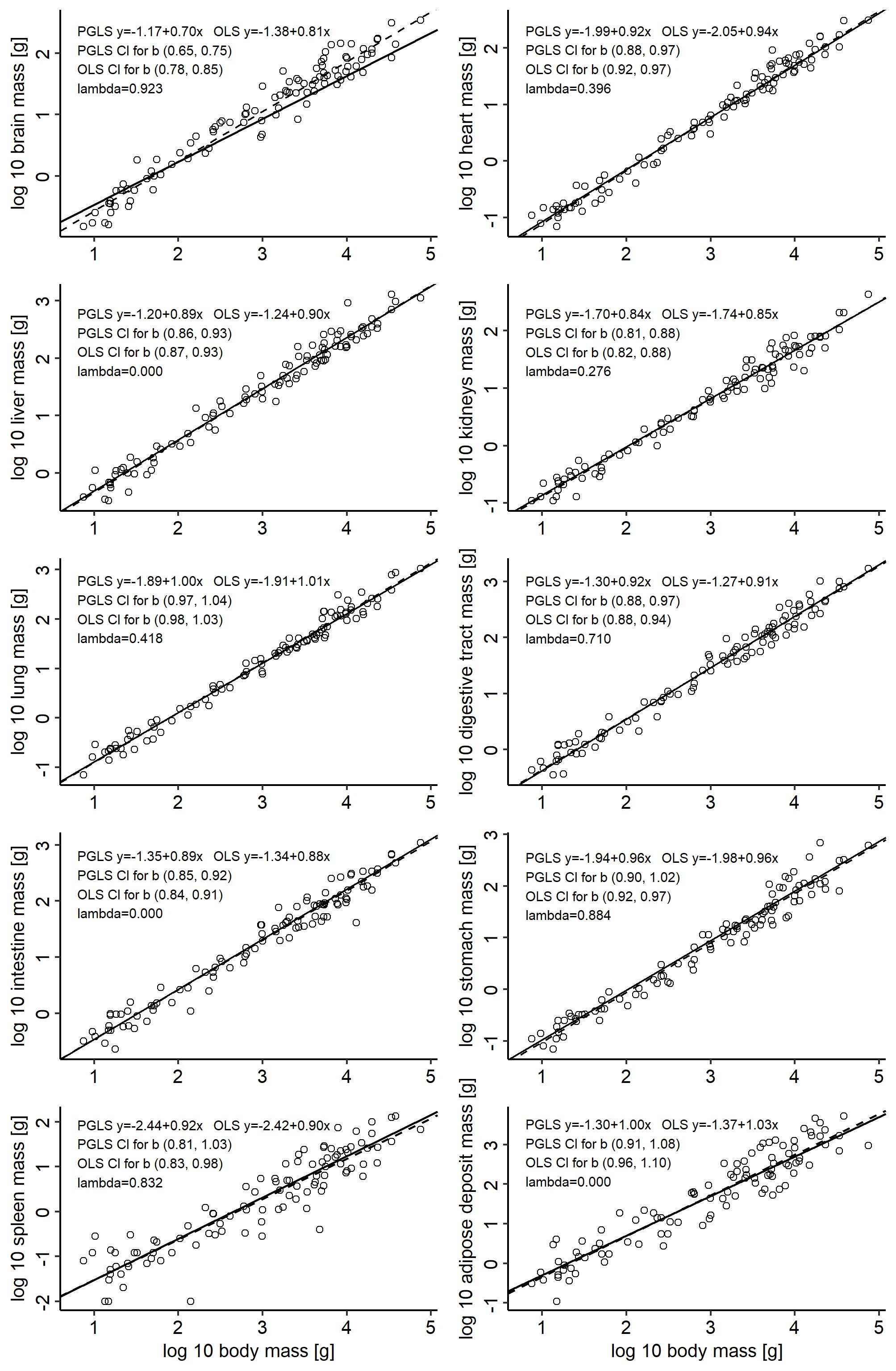


Figure S3. PGLS and OLS interspecific scaling of tissue/organ masses in mammals with log body mass as the independent variable.


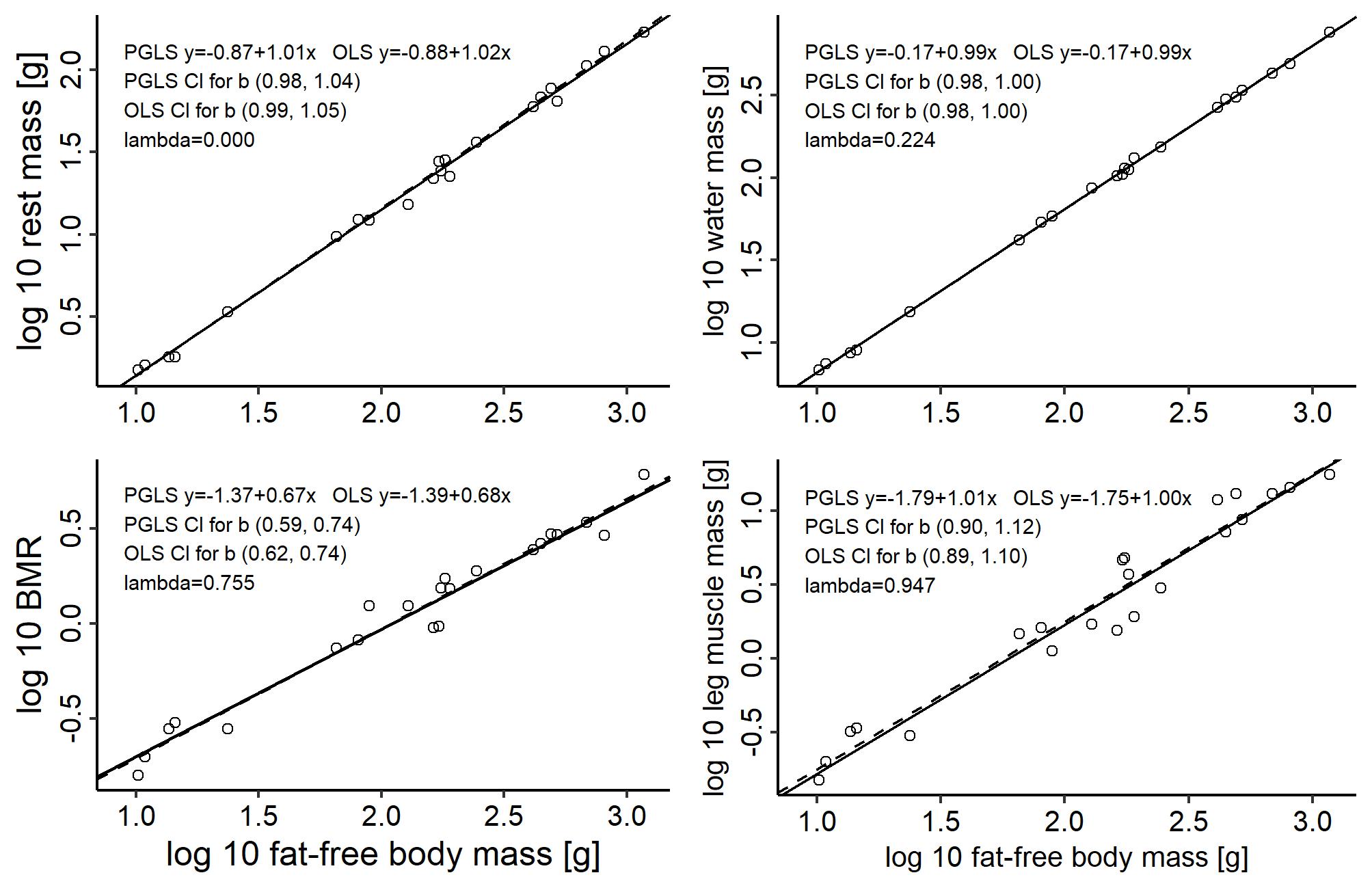


Figure S4. PGLS and OLS interspecific scaling of tissue/organ masses, water mass and BMR in birds with log10 fat-free body mass as the independent variable.


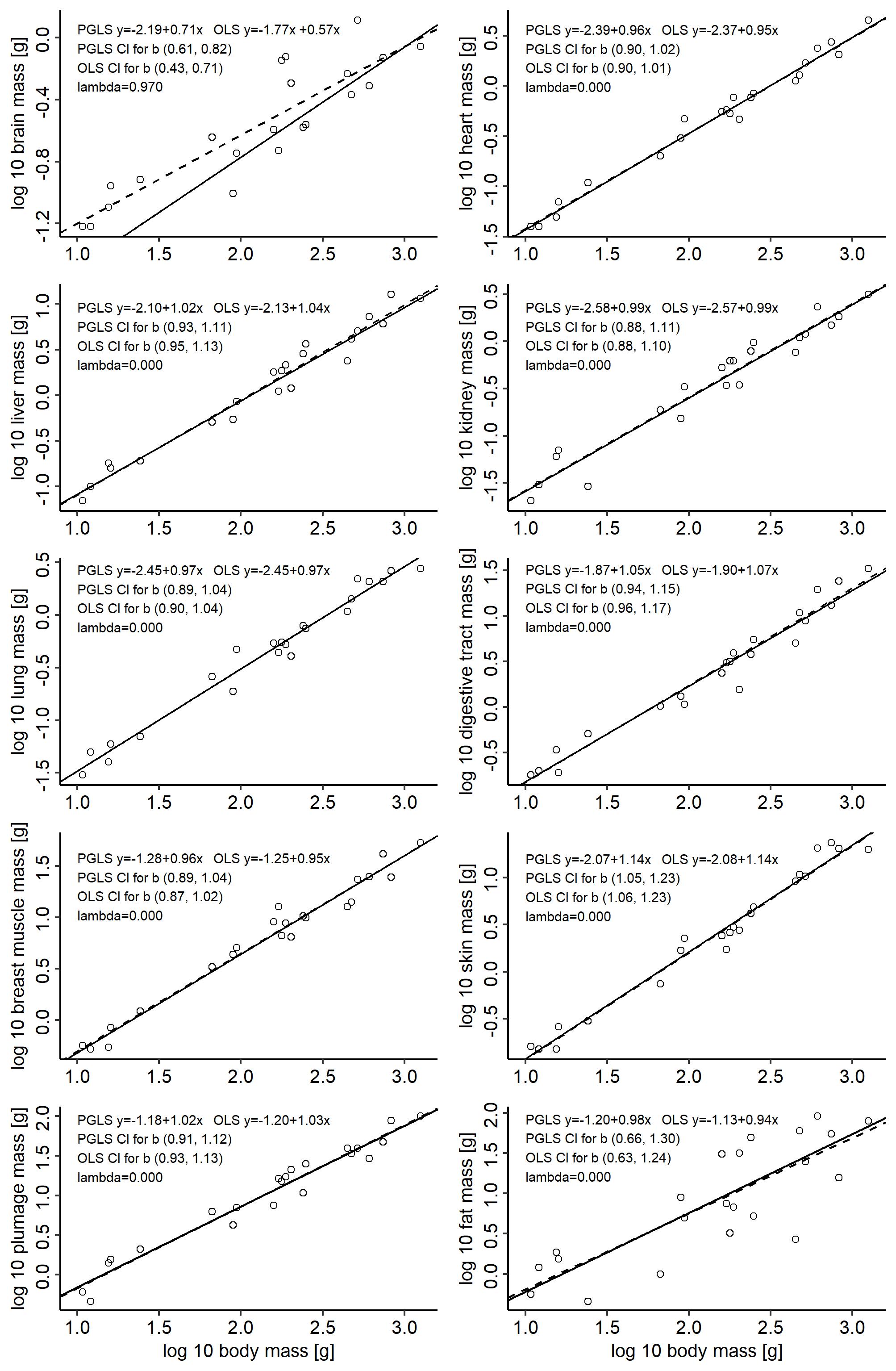


Figure S5. PGLS and OLS interspecific scaling of tissue/organ masses, water mass and BMR in birds with log body mass as the independent variable.
